# Diverse autophagy and apoptosis in myeloid leukemia cells induced by 20(s)-GRh2 and blue LED irradiation

**DOI:** 10.1039/c9ra08049j

**Published:** 2019-11-28

**Authors:** Jianjian Zhuang, Juxin Yin, Chaojian Xu, Mengmeng Jiang, Shaowu Lv

**Affiliations:** Key Laboratory for Molecular Enzymology and Engineering of the Ministry of Education, College of Life Science, Jilin University Changchun 130000 China lvsw@jlu.edu.cn; Research Centre for Analytical Instrumentation, Institute of Cyber-Systems and Control, State Key Laboratory of Industrial Control Technology, Zhejiang University Hangzhou Zhejiang Province 310058 P. R. China

## Abstract

Autophagy is an important mechanism for cell death regulation. To improve the anticancer effect during the treatment of leukemia and promote the apoptosis of leukemic cells, it is important to define the relationship between autophagy and apoptosis. A key bioactive compound in traditional Chinese medicine, 20(s)-Ginsenoside (GRh2), demonstrated an advancement in leukemia treatment. Blue LED therapy (BL) is a physical treatment method that can induce leukemic cell death. In this study, we tested the effect of 20(s)-GRh2, BL, and their combination (BL–GRh2) on the activation of leukemic cell apoptosis and autophagy. Both treatments, whether used individually or simultaneously, induce apoptosis through the induction of reactive oxygen species (ROS), disrupted mitochondrial membrane potential (MMP) and regulated the expression of apoptosis-related genes and proteins. Furthermore, using western blotting to analyze the autophagy markers LC3B and P62, we detected the activation of autophagy. In cells treated with autophagy inhibitor 3-MA, both autophagy and apoptosis were inhibited, either by BL alone or by BL–GRh2. However, apoptosis in 20(s)-GRh2-treated cells was enhanced. In cells treated with apoptosis suppressor Z-VAD-FMK, autophagy was inhibited in the BL and BL–GRh2-treated cells, although it was enhanced in cells treated with 20(s)-GRh2 alone. Moreover, we observed a stronger induction of apoptosis by BL–GRh2 in myeloid leukemia cells. Our data indicate that autophagy induced by different factors can play diverse roles on the same cells. Our results also indicate that the combination of traditional Chinese medicine with physical therapy may be a new strategy for anti-cancer therapy.

## Abstract 2

Blue LED and 20(S)-GRh2 induced apoptosis and autophagy in cells.

## Graphical abstract

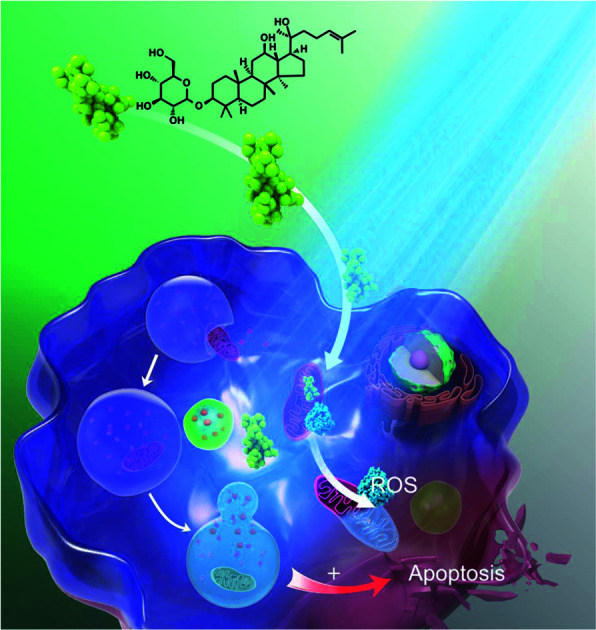

## 1. Introduction

Autophagy is a survival process that can (in certain specific paradigms) be converted to a cell death process which controls the proliferation of tumor cells and is related to many human diseases.^1–3^ Under specific conditions, autophagy leads to activation of programmed cell death due to the excessive degradation of molecules that are critical for cell survival and intracellular organelles, including mitochondria^4,5^. The apoptosis and autophagy interaction varies depending on cell type and conditions of stress, and can influence cancer cell destiny during anticancer therapies.^6^ Three main categories of interaction between apoptosis and autophagy were demonstrated. Firstly, autophagy can promote apoptosis.^7^ For instance, Park *et al.* proved that 3-MA (a specific autophagy inhibitor) can block apoptosis in nci-h460 cells.^8^ Secondly, autophagy can inhibit apoptosis, although the effect seems mutually dependent. It was demonstrated that specific anticancer pro-apoptotic drugs can increase the level of autophagy, while the autophagy inhibitors can increase tumor cell apoptosis.^9^ Finally, apoptosis and autophagy were shown to facilitate the cancer cell death synergistically.^10^

Leukemia is one of the world's deadliest types of malignancy.^11^ Among blood cancers, acute myeloid leukemia (AML) is a heterogeneous disease which can affect adults and children and has significant challenges clinically.^12^ Chronic myeloid leukemia (CML) is another blood cancer that requires long-term medical treatment, and the CML average survival time is only four years.^13^ Autophagy was determined as a key factor of leukemic cell responses to treatment. Yu *et al.* showed that autophagy could protect CML cells and maintain cell survival.^14^ Alternatively, it could also induce apoptosis of lymphoid leukemia cells.^15^ Diversity of responses suggests that autophagy induced by different factors can activate heterogeneous mechanisms and various targets in leukemia cells.

At present, the main treatment option for leukemia patients is chemotherapy, which is inevitably accompanied by adverse side effects in normal cells. Thus, development of novel methods of leukemia treatment are needed. Among recently explored methods, BL therapy is a non-invasive biophysical method which has been successfully used in clinical practice as a treatment for several diseases.^16–18^ Blue LED can induce apoptosis and autophagy in various cells. It can dose-dependently generate more ROS in OSC2 cells than in normal epithelial cells.^19^ Kuse *et al.* reported that BL can convert the autophagy marker LC3-I into LC3-II in mouse-derived 661 W cells indicating the activation of autophagy.^20^

Ginsenosides are the main active chemical constituents of ginseng,^21^ which have great pharmacological efficacy and play an important role in anti-fatigue, anti-aging and anti-tumor.^22,23^ Accumulating evidence indicated that ginsenoside can also induce autophagy,^24–26^ which is essential for the degradation and recycling of cellular constituents. Ginsenoside Rh2 (GRh2), identified as a protopanaxadiol-type ginsenoside, which exists in two stereoisomeric forms, 20(R)-GRh2 and 20(S)-GRh2. Compared with 20(R)-GRh2,^27^20(S)-GRh2 has been widely researched and displays an more evident anti-cancer activity. Recent studies have found that 20(S)-GRh2 can inhibit the proliferation of leukemic KG1-α and K562 cells.^28^ Our previous study also demonstrated that 20(S)-GRh2 can induce autophagy which has a cooperative manner with apoptosis in U937 and K562 cells.^29^

In this study, we demonstrated that autophagy induced by traditional Chinese medicine (20(s)-GRh2) alone or combined with physical factor (BL) has different impacts on apoptosis in AML and CML leukemic cells. Autophagy and apoptosis induced by combined BL–GRh2 treatment and BL alone indicated mutually reinforcing effects. Simultaneously, pronounced changes in apoptosis and autophagy related proteins and genes were observed. Furthermore, combined BL–GRh2 treatment demonstrated a better cancer-killing effect in K562 and U937 leukemia cells.

## 2. Experimental

### 2.1. Materials and LED irradiation setup

BCA and CCK-8 Assay kits as well as Hoechst Staining and Caspase-3 Activity Assay Kits were received from BestBio, China. An MDC staining kit, FBS, 3-MA and Z-VAD-FMK were received from Sigma. An RPMI 1640 medium was obtained from Gibco, and an ROS detection kit was obtained from Beyotime. Caspase-9 and MMP detection kits were received from KeyGen, China. The entire qRT-PCR kit was obtained from Takara. All antibodies were purchased from CST 9661. A BL photoreactor was home-made and consisted of 36 LED arrays and a 12 V battery, with an intensity of 0.25 mW cm^−2^.

### 2.2. Cell culture and treatment

The RPMI 1640 medium with streptomycin and 10% fetal bovine serum (FBS) were used to culture U937 cells (ATCC CRL 1593) and the K562 cells (ATCC CCL 243) at 37 °C, 5% CO_2_. The K562 and U937 cell lines were maintained at a density of 1 × 10^5^ cells per mL. Cells were treated with BL (2 h), GRh2 (40 μM or 60 μM), BL + GRh2 (2 h + 40 μM or 2 h + 60 μM). The control group was constituted with cells in 0.5% DMSO medium.

### 2.3. Cell viability

For cell viability assay, 1 × 10^4^ cells per well were seeded in 96-well plates at 37 °C in a 5% CO_2_ humidified atmosphere. Cells were treated with BL (2 h), GRh2 (40 μM or 60 μM), BL + GRh2 (2 h + 40 μM or 2 h + 60 μM) for 24 h. After treatment, CCK-8 assay was used to assess cell's viability under different factors. Incubation was continued for four hours at 37 °C, 5% CO_2_. Cells were rinsed with PBS and dyed with Hoechst for 0.5 hours. Absorbance at 450 nm was detected using a microplate reader.

### 2.4. Apoptosis analysis

The cells were seeded at a density of 5 × 10^5^ cells per well in 6-well plates. The cells were treated with various conditions. After treatment, Cells were then dyed with Hoechst 33342 staining solution for 30 minutes to observe apoptotic cells at specific time intervals. The Annexin V-FITC method was employed to analyze cells' apoptosis rates. Cells were treated with annexin V binding buffer (containing 0.4% FITC-Annexin V) for 15 minutes. The apoptosis ratios were analyzed with flow cytometry.

### 2.5. Detection of ROS and mitochondrial membrane potential (MMP)

The cells were seeded at a density of 5 × 10^5^ cells per well in 6-well plates. The cells were treated with various conditions. After treatment, ROS was determined using a H2DCFDA fluorescent probe. It was incubated at 37 °C for 0.5 hours when the H2DCFDA (10 mM) was added. For MMP detection, the differently treated cells were washed two or three times with cold PBS and stained with JC-1 in the dark at 37 °C for 0.5 hours. Fluorescence was measured by flow cytometry.

### 2.6. Quantitative reverse transcriptase PCR (RT-PCR)

The cells were seeded at a density of 5 × 10^5^ cells per well in 6-well plates. The cells were treated with various conditions. After treatment, qRT-PCR was used to detect gene expression. After cells had been treated, according to the procedure of the RNA extraction kit and Reverse Transcription kit, the cDNA was synthesized. The primer for qRT-PCR can be seen in Table 1. qRT-PCR was performed on the ABI 7500 with SYBR kit and results were analyzed with equation 2^−ΔΔ*C*_t_^.

**Table 1:** Primer sequences

Gene	Forward primer	Reverse primer
bax	5′GGAGGAAGTCCAATGTCCAG3′	5′ GGGTTGTCGCCCTTTTCTAC3′
bcl-2	5′GAGAAATCAAACAGAGGCCG3′	5′ CTGAGTACCTGAACCGGCA3′
bcl-xl	5′ CTGCTGCATTGTTCCCATAG 3′	5′ TTCAGTGACCTGACATCCCA 3′
Caspase3	5′ CTGCCTCTTCCCCCATTCT 3′	5′ TCGCTTCCATGTATGATCTTTG 3′
Caspase9	5′ AGGTTCTCAGACCGGAAACA 3′	5′ CTGCATTTCCCCTCAAACTC 3′
Beclin-1	5′ CTCCTGGG TCTCTCCTGGTT3′	5′ TGGACACGAGTTTCAAGATCC3′
ATG5	5′ GCCATCAATCGGAAACTCAT 3′	5′ ACTGTCCATCTGCAGCCAC 3′
LC3B	5′GAGAAGACCTTCAAGCAGCG 3′	5′ TATCACCGGGATTTTGGTTG 3′
ATG7	5′ ATTGCTGCATCAAGAAACCC 3′	5′ GAGAAGTCAGCCCCACAGC3′
β-Actin	5′ TGACGTGGACATCCGCAAAG 3′	5′ CTGGAAGGTGGACAGCGAGG 3′

### 2.7. Activity determination of caspase-3 and caspase-9

The cells were seeded at a density of 5 × 10^5^ cells per well in 6-well plates. The cells were treated with various conditions. After treatment, the activity was measured following the procedure in the activity detection kit. Cells in different groups were washed three times with cold PBS. When the lysis buffer was added, the protein was released and combined with its specific substrate, labeled by the fluorescence. The fluorescent plate reader was used to detect the activation intensity of caspase-9 and caspase-3 proteins.

### 2.8. Autophagy observation

The cells were seeded at a density of 5 × 10^5^ cells per well in 6-well plates. The cells were treated with various conditions. After treatment, MDC was used to stain differently treated cells and an autophagy phenomenon was observed by the fluorescence microscope (excitation: 355 nm emission: 512 nm). For further observation of autophagy, a TEM assay was performed. Cells were pretreated according to the methods of a previous study.^30^ Cells were rinsed with PBS and 2.5% phosphate-buffered glutaraldehyde was used to fix cells at 4 °C for one hour. After washing three times with PBS, 1% osmium tetroxide (OsO_4_) was used to the post-fixation process. Ethanol and acetone were used for dehydration and embedded in epoxy resin before being observed in EM 1400 at 8 × 10^4^ V.

### 2.9. Western blotting analysis

The cells were seeded at a density of 5 × 10^5^ cells per well in 6-well plates. The cells were treated with various conditions. After treatment, the proteins related to apoptosis and autophagy of myeloid leukemia cells were detected using western blot. Cells were lysed in RIPA buffer (1 mM PMSF) and centrifuged at 12000*g*. The supernatant was collected and the protein concentration measured with a BCA kit. Following this, 10% SDS-PAGE electrophoresis was used to separate the protein, after which the separated protein was dropped onto a PVDF membrane. PBST (5% non-fat dry milk) was used to block the membranes and then incubated with an antibody overnight. The HRP-conjugated anti-rabbit antibody was added to incubated for two hours. Primary antibodies were used at the following dilutions: 1:1000 for anti-cleaved Caspase3 (Cell Signaling #9661), 1:1000 for PARP antibody (Cell Signaling #9542),1:1000 for β-actin antibody (Cell Signaling #4970),1:1000 for LC3B antibody (Cell Signaling #3868), 1:1000 for p62 antibody (Cell Signaling #8025); secondary antibodies were used at the following dilutions: 1:5000 for Anti-mouse IgG, HRP-linked Antibody (Cell Signaling #7076) and Anti-rabbit IgG, HRP-linked Antibody (Cell Signaling #7074).

### 2.10. Statistical analysis

Data are expressed as mean ± standard deviation (SD) and were analyzed with GraphPad software. Two-tailed *t*-tests were used to analyze statistical differences.

## 3. Results

### 3.1. Apoptosis induced by different factors in myeloid leukemia cells

**Fig. 1:**
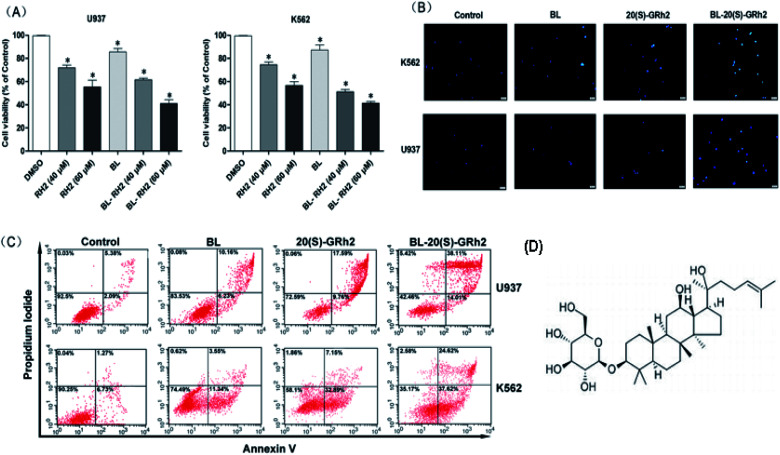
Chemical structure of GRh2 and Pro-apoptotic effects of BL, GRh2, BL–GRh2. (A) Hoechst 33342 staining of myeloid leukemia cells treated with GRh2, BL, and BL–GRh2. (B) Proliferation inhibition treated with BL, GRh2, and BL–GRh2 in myeloid leukemia cells. (C) Apoptotic ratio (early and late apoptosis) treated with BL, GRh2, and BL–GRh2. (D) Chemical structure of GRh2. The control group was treated with 0.5% DMSO and the *p* values are *p* < 0.05 for control group.

At first, we compared the proliferation inhibition effect of the BL, GRH2 and BL–GRH2 groups on myeloid leukemia cells. Results suggested that viability of myeloid leukemia cells in Fig. 1A was suppressed by BL–GRh2. Meanwhile, BL–GRh2 demonstrated a higher inhibition of proliferation compared with GRh2 group. When compared with the BL effect alone, this demonstrated almost 50% higher inhibition of proliferation. In order to verify the proposal method is safe normal human cells but show toxicity to cancer cells, we have already employed 2 h irradiation time to assess the effect of BL on the viability of PBMC and U937 cells in our previous study^31^ and we found that the same irradiation time cannot induce evident inhibition of proliferation in PBMC. Meanwhile, previous studies have also demonstrated that 20(S)-GRh2 (60/80 μM) exhibited low toxicity on non-cancerous cells (normal bone marrow).^32,33^ In the Annexin V-FITC assay, treatment with BL–GRh2 resulted in 52.12% of apoptosis, which was higher than with BL alone (16.39%) or 20(S)-GRh2 (27.35%) alone in U937 cells (Fig. 1B). Similarly in K562 cells, treatment with BL–GRh2 resulted in 62.24% apoptosis, which was higher than BL alone (14.89%) or 20(S)-GRh2 alone (40.04%). This indicated that combined therapy increased apoptosis in myeloid leukemia cells. Meanwhile, we observed apoptosis of myeloid leukemia cells by Hoechst staining. The BL–GRh2 group demonstrated a more significant chromatin condensation and nuclear fragmentation (Fig. 1C). Furthermore, we used flow cytometry to determine the apoptosis ratio.

### 3.2. Mitochondrial apoptosis induced by different factors in myeloid leukemia cells

**Fig. 2:**
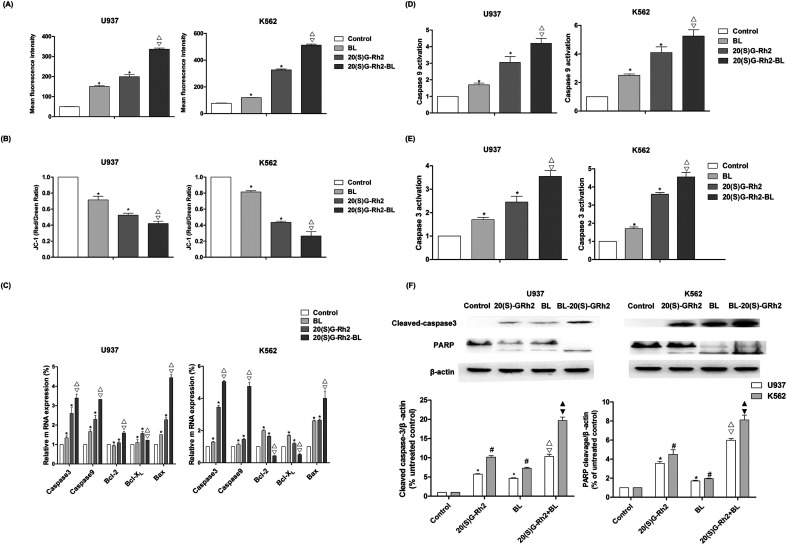
Mitochondrial apoptosis induced by BL, GRh2, BL–GRh2 in myeloid leukemia cells. (A) The level of ROS induced by BL, GRh2, BL–GRh2. ROS levels are expressed using fluorescence intensity. (B) Changes to MMP in different groups. Flow cytometry was used to analyze the fluorescence of JC-1. (C) The expression of apoptosis related genes was determined by qRT-PCR. The qRT-PCR was performed using the Prism 7500 Sequence Detection System; (D and E) The activity of caspase-9 and caspase-3 was measured by using a protein activity assay; and, (F) western blotting technique detected the effect of different group on the apoptosis-related proteins (caspase-3, PARP). The loading control protein is β-actin. Results of three experiments are shown as the mean ± SD. *P values are *p* < 0.05 for control group; * *p values are *p* < 0.001 for control group; ^△^*p* values are *p* < 0.05 for BL-treated cells; ^▽^*p* values are *p* < 0.05 for GRh2-treated cells.

ROS is an important signaling factor for apoptosis. Treatment of myeloid leukemia cells with different factors increased ROS levels. Moreover, the level of ROS in BL–GR2 group is higher than the GRh2 or BL groups (Fig. 2A). Disruption of MMP (Fig. 2B) was also observed in different groups. The relative fluorescence intensity induced by the BL–GRh2 treatment (0.42) was lower than that of BL treatment (0.69) or GRh2 treatment (0.79) in U937 cells. Likewise, in K562 cells, the BL–GRh2-induced fluorescence intensity (0.26) was lower than in the cells treated by BL alone (0.81) or 20(S)-GRh2 alone (0.43). Bcl-2, bcl-xl was an anti-apoptotic gene and bax was a pro-apoptotic gene which is able to control the MMP. Results in Fig. 2C show that bax/bcl-xl and bax/bcl-2 in myeloid leukemia cells treated by BL–GRh2 is higher than the ratios in the BL group and GRh2-treated cells. Meanwhile, ratios in the GRh2 group are higher than in the BL group. The pro-apoptosis protein can be furthered controlled by increased ratios, and the increased caspase-3 and caspase-9 were also observed (Fig. 2D and E). Meanwhile, the activity of caspase-9 and caspase-3 was higher than in the BL or GRh2 groups, while the GRh2 group had higher levels of myeloid leukemia cells than the BL group. Furthermore, the western blot also suggested an increased cleavage of caspase-3 and PARP (Fig. 2F). Our results indicate that apoptosis was initiated *via* the mitochondrial pathway, findings which are consistent with the data found during testing of proliferation.

### 3.3. Autophagy induced by different factors in myeloid leukemia cells

**Fig. 3:**
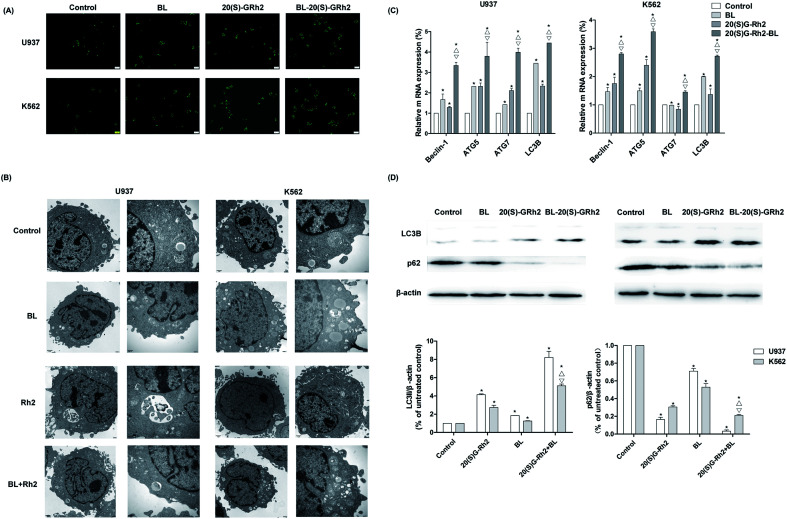
Autophagy induced by BL, GRh2, BL–GRh2 in myeloid leukemia cells. (A) Autophagic vacuoles in different groups observed using MDC staining (Bar = 20 μm). (B) Autophagic vacuoles observed by TEM when treated with different factors. (C) Detection of expression levels of autophagy-related genes. (D) Western blot analysis of the LC3B and p62 when treated with different factors. Results are from three experiments and are shown as the mean ± SD. ^★^*P* values are *p* < 0.05 for control cells; △ *p* values are *p* < 0.05 for BL-treated group; ▽ *p* values are *p* < 0.05 for GRh2 groups.

Autophagy induced by BL, GRh2 and BL–GRh2 in myeloid leukemia cells was first observed by MDC staining. The acidic vesicular organelles can be seen in Fig. 3A. Moreover, TEM results demonstrate significant autophagic vacuoles in myeloid leukemia cells when treated with different factors (Fig. 3B). These results suggest that BL, GRh2 and BL–GRh2 can induce the production of autophagy. Through further detection of autophagy-related genes, we found that the BL–GRh2 group can more significantly induce the expression of ATG5, ATG7, Beclin1 and LC3B and that the expression of the BL and GRh2 groups are also different. Meanwhile, results in Fig. 3D demonstrate that the BL–GRh2 treatment of myeloid leukemia cells induces increased conversion from LC3-I to LC3-II; this is more so than BL or 20(S)-GRh2. In addition, the conversion in GRh2 group is higher than BL group. It also show that the BL–GRh2 treatment decreased the expression of p62 protein less than BL or 20(S)-GRh2, while the expression is lower in the GRh2 group than in the BL group. These results indicate that BL–GRh2 could induce more significant autophagy than GRh2 and BL. Meanwhile, GRh2 could induce more significant autophagy than BL.

### 3.4. Apoptosis and autophagy when the autophagy was inhibited in different groups

**Fig. 4:**
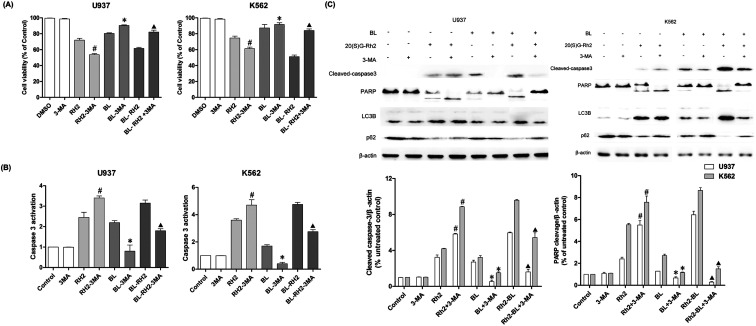
Apoptosis variation when autophagy was inhibited in different treated groups (BL, GRH2, BL–GRH2). (A) Cell viability when pretreated with 3-MA in different groups. (B) Changes in caspase3 activation when pretreated with 3-MA in different groups. (C) Western blot analysis of changes in apoptosis and autophagy. Results are from three experiments and are shown as mean ± SD. #*P* < 0.05 for Rh2 treated cells; *p < 0.05 for BL treated cells; ▲*p* < 0.05 for BL–Rh2 treated cells.

We used autophagy inhibitors to observe the effect of autophagy on apoptosis when treating myeloid leukemia cells with BL, GRh2, BL–GRh2. In the BL–GRh2 and BL groups, the proliferation inhibition of myeloid leukemia cells was decreased when the autophagy was inhibited by 3-MA. Moreover, decreased activation of caspase-3 in myeloid leukemia cells can also be observed in Fig. 4B. Fig. 4C displays decreased cleaved-caspase3 levels as well as inhibited PARP cleavage when autophagy was inhibited. Furthermore, decreased conversion of LC3B and increased p62 in myeloid leukemia cells can also be observed. These results demonstrate that the apoptosis was inhibited when autophagy inhibited. However, in the GRh2 group, this phenomenon is reversed.

### 3.5. Autophagy and autophagy when the apoptosis was inhibited in different groups

**Fig. 5:**
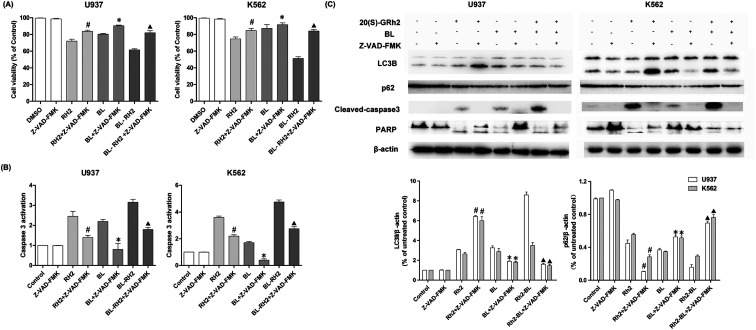
Autophagy variation when apoptosis was inhibited in different treated groups. (A) Cell viability when pretreated with Z-VAD-FMK in different groups. (B) Changes to caspase3 activation when pretreated with Z-VAD-FMK in different groups. (C) Western blot analysis of changes in apoptosis and autophagy. Results are from three experiments and are shown as mean ± SD. #*P* < 0.05 for Rh2 treated cells; *p < 0.05 for BL; ▲*p* < 0.05 for BL–Rh2 treated cells.

We used Z-VAD-FMK to inhibit apoptosis and observe changes in autophagy in different treatment groups (BL, GRh2 and BL–GRh2). Results demonstrate that cell viability can be recovered in the three groups when Z-VAD-FMK is added. Meanwhile, decreased activation of caspase-3 in myeloid leukemia cells can be observed in Fig. 5B. These results demonstrate that apoptosis was inhibited. Furthermore, in the BL–GRh2 and BL groups, decreased conversion of LC3B and increased p62 can be found, which suggests that autophagy was inhibited when the apoptosis was inhibited. However, in the GRh2 group, this phenomenon reversed.

## 4. Discussion

BL and GRh2 have anti-cancer effects and lead to very low toxicity in typical cells, suggesting that these treatment options should be considered for leukemia.^18,25^ They represent two different approaches in the treatment of leukemia. We proposed that leukemic cell apoptosis and anticancer treatment can be promoted by specific activation of autophagy. Accordingly, understanding the effect of autophagy on apoptosis is of great significance as it can make treatment more effective.^2,29,34–36^ In this study, we investigated the difference of autophagy induced by BL, GRh2 and BL–GRh2 in myeloid leukemia cells. We observed a complex influence of autophagy on apoptosis induced by 20(S)-GRh2, BL, and BL–GRh2 therapy. Meanwhile, BL–GRh2, a more effective therapy for myeloid leukemia, was proposed.

Apoptotic cells are marked by increased cell membrane permeability that can be detected through annexin-V staining.^37^ We used Hoechst 33342 staining and annexin-V flow cytometry to investigate apoptosis. We also observed typical morphological features of apoptotic cells. As shown in Fig. 1B, BL–GRh2 therapy exhibited the most effective level of growth inhibition in myeloid leukemia cells compared with BL or GRh2 treatments alone. A higher percentage of apoptotic myeloid leukemia cells was observed following treatment with BL–GRh2. We have demonstrated that GRh2 and BL can induce the release of ROS. From the physical point of view, the enzymes which contain porphyrin in mitochondria are proposed as acceptors for BL irradiation,^38,39^ which would produce a large amount of ROS. ROS release from neighboring mitochondria, increase the permeation of mitochondrial membrane and further lead to depolarization of the mitochondrial membrane potential which is widely considered as one of the earliest events in the process of apoptosis.^19,40–43^ On the other hand, excessive ROS can come into the nucleus and cause DNA damage to induce genetic mutations.^44^ In addition, the ROS also could indiscriminately react with biomacromolecules such as lipids and protein in cells to induce oxidative damage.^45^ In our study, we only focus on the first effect induced by ROS. Our results indicated that the level of ROS was higher (Fig. 2A), while MMP was lower (Fig. 2B) following treatment with BL–GRh2 when compared with GRh2 or BL treatments alone. The increased ratio of bax/bcl-2(bcl-xl) can control the MMP.^46,47^ In Fig. 2C, a higher ratio of those proteins was achieved following treatment by BL–GRh2 when compared with GRh2 or BL treatments alone, a finding which was consistent with the observed changes in MMP. Pro-apoptotic factors, caspase-9 and caspase-3 can be further released.^28^ Activated caspase-3 can cleave specific substrates such as PARP, leading to irreversible cell death. The results in Fig. 2C–F show that gene and protein expressions in myeloid leukemia cells are higher than when treated with BL–GRh2. These results suggest that BL–GRh2 may be a more effective therapy for myeloid leukemia.

Our research indicated that autophagy can be induced by BL, GRh2 and BL–GRh2 in myeloid leukemia cells. Ultrastructural features of autophagy can be found in Fig. 3A and B.^48^ Studies have demonstrated that increased levels of beclin-1, ATG7 and ATG5 can be found when autophagy is enhanced.^49,50^ Our results (Fig. 3C) are consistent with these findings. It is well known that autophagy is positively related to levels of LC3-II/I and negatively related to levels of P62.^51^ We also found that autophagy is higher in cells which have been treated with BL–GRh2 compared with GRh2 or BL treatments alone.

The effect of autophagy on apoptosis are complex and unclear.^52^ It could be a result of a partner, antagonist, or promoter of apoptosis.^53^ In order to find out whether the autophagy induced by these two factors is different, we treated myeloid leukemia cells with autophagy inhibitor 3-MA. Results indicated that when autophagy was inhibited, the apoptosis of BL and BL–GRh2 group was also inhibited (Fig. 4). However, increased apoptosis can also be found in the GRh2-treated group. Z-VAD-FMK, an apoptosis inhibitor, was used to assess autophagy under conditions of blocked cell death signaling. In the BL and BL–GRh2 groups, results suggested that when apoptosis was decreased (Fig. 5), autophagy was inhibited. Likewise, increased autophagy can be found in cells treated with GRh2. Consequently, we propose that autophagy induced by different factors in the same cells may have different effects on apoptosis. Autophagy and apoptosis induced by BL–GRh2 or BL may be mutually reinforcing and may promote irreversible cancer cell death.

From chemical point of view, the specific structural of 20(S)–Rh2 make it possess cell biology effects. The previous study has reported that the stereochemistry of the hydroxyl group at C-20 of 20(S)–Rh2 manifest antiproliferative action on prostate cancer cells.^54^ Park *et al.* have reported Rh2-induced apoptosis in hepatoma cells dependent on the active of ROS.^55^ In our work, we also found an increase in ROS levels. From physical point of view, cellular components such as flavins and porphyrin ring structures serve as endogenous photoactivators which absorb blue light and increase the level of intracellular ROS.^38,39^ Moreover, we also observed this phenomenon. The signal transduction pathways of autophagy and apoptotic induced by BL irradiation has not yet been clarified. In the future research, we will further study the mechanism of redox enzyme regulated the apoptosis and autophagy under the physics and chemical condition.

## 5. Conclusion

In conclusion, our study demonstrated that autophagy which has been induced by different factors plays diverse roles in leukemic cells and has provided important new insights into our understanding of the role of autophagy in different factors-induced apoptosis. We found that the combined 20(S)-GRh2 and BL (BL–GRh2) therapy has a stronger death-promoting effect on U937 and K562 cells compared to 20(S)-GRh2 and BL treatments alone. Combined BL–GRh2 treatment facilitated mutually reinforcing interaction between autophagy and apoptosis, resulting in enhanced cancer cell death. In future studies, we will clarify the underlying mechanisms of the observed phenomenon.

## Author contributions

Jianjian Zhuang, Juxin Yin, and Shaowu Lv designed the experiments; Juxin Yin, Jianjian Zhuang, Mengmeng Jiang and Chaojian Xu performed the experiments; Jianjian Zhuang, Juxin Yin and Chaojian Xu analysed the data; Jianjian Zhuang, Juxin Yin,and Shaowu Lv wrote the paper; and all the authors reviewed the manuscript.

## Data availability statement

The datasets used or analysed during the current study are available from the corresponding author on reasonable request.

## Conflicts of interest

The authors declare no conflict of interest.

## Supplementary Material
